# Ultrasonic-Assisted Dual-Alkali Pretreatment and Enzymatic Hydrolysis of Sugarcane Bagasse Followed by *Candida tropicalis* Fermentation to Produce Xylitol

**DOI:** 10.3389/fnut.2022.913106

**Published:** 2022-05-18

**Authors:** Lili Gai, Er-Fang Ren, Wen Tian, Debao Niu, Weidong Sun, Fangxue Hang, Kai Li

**Affiliations:** ^1^College of Light Industry and Food Engineering, Guangxi University, Nanning, China; ^2^Guangxi Subtropical Crops Research Institute, Nanning, China

**Keywords:** ultrasonic, enzymatic hydrolysis, sugarcane bagasse, xylitol, pretreatment

## Abstract

In this work, the investigation mainly focused on ultrasonic-assisted dual-alkali pretreatment and enzymatic hydrolysis of sugarcane bagasse followed by *Candida tropicalis* fermentation to produce xylitol. The results showed that the combination of NaOH and ammonia water had the best effect by comparing the effects of the four single-alkali (NaOH, KOH, ammonia water, Ca(OH)_2_) and their mixed double-alkali pretreatments on xylose content. Then, the optimal conditions for ultrasonic-assisted pretreatment and enzymatic hydrolysis of sugarcane bagasse were obtained by response surface methodology. When the ratio of NaOH and ammonia water was 2:1, the mixed alkali concentration (v/v) was 17%, the ultrasonic temperature was 45°C, the ultrasonic power was 300 W, and the ultrasonic time was 40 min, the content of xylose reached a maximum of 2.431 g/L. Scanning electron microscopy showed that sugarcane bagasse by ultrasonic-assisted alkali pretreatment aggravated with more folds and furrows. Moreover, the fermentation results showed that the concentration ratio of enzymatic hydrolysate of sugarcane bagasse affected the xylitol yield, and when concentrated three times, the highest yield of xylitol (54.42%) was obtained.

## Introduction

As one of the main by-products of sugarcane processing to produce sugar, sugarcane bagasse is usually burned or treated as soil fertilizer, resulting in low added value due to its unreasonable disposal (1). The major components of sugarcane bagasse are cellulose, hemicellulose, and lignin, of which hemicellulose is a good source of xylose and can be further hydrolyzed into xylitol by microbial fermentation and enzymatic conversion (2, 3). Xylitol is a natural sweetener extracted from birch, oak, corncob, sugarcane bagasse, and other plant raw materials, which are also widely used in the food industry as food additives (4). Xylitol can be produced using microbial fermentation. The *Candida tropicalis* has a strong xylose conversion ability and high tolerance to inhibitors such as furfural, acetate, and phenolics in xylose mother liquor (5).

Pretreatment of lignocellulosic biomass is a critical step in xylitol production. The common methods of pretreatment of sugarcane bagasse are physical methods, chemical methods, biological methods, and physical-chemical combination methods. Common physical methods such as mechanical activation (6), microwave treatment (7), and ultrasonic treatment (8) mainly increase the surface area of the raw material by changing the physical structure of the lignocellulosic raw materials, while chemical methods mainly use chemical reagents such as acid, alkali, and ionic liquid to make raw materials swell, thereby disrupting the tightly bound structure of hemicellulose, lignin, and cellulose, reducing the crystallinity of cellulose (9). Alkaline pretreatments, including NaOH, Ca(OH)_2_, and ammonia water pretreatment, can be used to remove lignin from sugarcane bagasse, which generally swells the sugarcane bagasse and reduces the crystallinity of cellulose (10–12). Besides, biological pretreatment is performed by enzymes or fungi during the raw material storage, and hemicellulose and lignin are partially removed (13). However, the biological pretreatment is still in the theoretical stage and cannot be put into practical production due to its time-consuming and high requirements for reaction conditions.

As a non-thermal food processing technique, ultrasound has been widely applied in various fields of food processing such as crystallization (14), hydrolysis (15), extraction (16), and microbial inactivation. Compared with the conventional thermal techniques, the ultrasound technique presents many advantages such as no chemical residues, minimal thermal effects, and less influence on food quality (17). The effect of ultrasonic on lignocellulosic is mainly derived from mechanical action and cavitation, while ultrasound pretreatment can disrupt cell wall structure to increase the available surface area of biomass. Previous studies have shown that ultrasonic pretreatment combined with chemical methods can improve reaction efficiency and achieve efficient lignin removal (18, 19). Therefore, ultrasonic-assisted alkali pretreatment could be a novel technique to promote the enzymatic hydrolysis of sugarcane bagasse.

The objective of this study was to investigate the effects of ultrasonic-assisted four single-alkali (NaOH, KOH, ammonia water, Ca(OH)_2_) and their mixed double-alkali pretreatments on the enzymatic hydrolysis of sugarcane bagasse, and to further evaluate the effect of the concentration ratio of the obtained enzymatic hydrolysate on the production of xylitol by *Candida tropicalis* fermentation.

## Materials and Methods

### Materials and Reagents

Sugarcane bagasse was obtained from Guangxi Baiguitang Food Science and Technology Co., Ltd. (Guigang, China) and passed through the 80-mesh screen to obtain the powder for further use. Sodium hydroxide, potassium hydroxide, calcium hydroxide, and ammonia water were from Damao Chemical Reagent Factory (Tianjin, China); xylanase was sourced from Sigma-Aldrich (San Francisco, USA); *Candida tropicalis* 31949 was purchased from China Industrial Microbiology collection management center (Beijing, China). All reagents were of analytical grade unless otherwise mentioned.

### Ultrasonic Assisted Alkali Pretreatment

The numeric control ultrasonic cleaning machine (KQ-500DE, Kunshan ultrasonic instruments Co. Ltd., Kunshan, China) was applied in this work. One gram sugarcane bagasse powder and 20 mL NaOH solution (10%) were mixed in a 100 mL conical flask. The treated temperature of the treatment was 60°C and the power of the ultrasonic was 300 W for 50 min. After the ultrasonic treatment, the pH of the mixture solution was adjusted to 5.5, and 0.05 g xylanase was added to the constant temperature water bath oscillator to obtain xylose. The xylose content was measured using the phloroglucinol method with xylose as the reference with a slight modification (20).

Among them, the alkali solution conditions were changed as follows: 20 mL 10% NaOH solution (A); 20 mL 10% KOH solution (B); 20 mL 10% ammonia water (C); 20 mL 10% Ca (OH)_2_ solution (D); 10 mL 10% NaOH solution and 10 mL 10% KOH solution (AB and BA); 10 mL 10% NaOH solution and 10 mL 10% ammonia water (AC and CA); 10 mL 10% NaOH solution and 10 mL 10% Ca (OH)_2_ solution (AD and DA); 10 mL 10% KOH solution and 10 mL 10% ammonia water (BC and CB); 10 mL 10% KOH solution and 10 mL 10% Ca (OH)_2_ solution (BD and DB); 10 mL 10% ammonia water and 10 mL 10% Ca (OH)_2_ solution (CD and DC).

### Morphological Property

Scanning electron micrographs (SEM) were taken by a scanning electron microscope (F16502, Phenom, Netherlands). All samples were subsequently coated with a thin gold layer before examining under a scanning electron microscope.

### Experimental Design and Statistical Analysis

To optimize the preparation conditions and obtain the maximum xylose content, the Box–Behnken experimental design (BBD) of the response surface methodology (RSM) was carried out with five variables at each of the three levels. The most important key independent variables and their optimization ranges, such as the ratio of NaOH and ammonia water (A: 1:1, 2:1, 3:1); mixed alkali concentration (B: 10, 15, 20%, v/v); ultrasonic temperature (C: 30, 40, 50°C); ultrasonic power (D: 200, 300, 400 W) and ultrasonic time (E: 20, 30, 40 min). Forty-six combinations were produced by the BBD as shown in Table 1. The design was analyzed using Design Expert 8.0 for the experiments. All determinations were conducted in triplicate.

**Table 1:** Box-behnken design (BBD) matrix with response values for the xylose content.

**Run**	**A**	**B**	**C**	**D**	**E**	**Response**
1	1:1	15	30	300	30	1.486
2	3:1	10	40	300	30	1.589
3	2:1	20	40	400	30	1.287
4	3:1	15	40	300	40	1.924
5	2:1	15	50	300	20	2.072
6	3:1	15	40	400	30	1.367
7	2:1	10	40	400	30	1.295
8	2:1	15	50	300	40	2.141
9	2:1	20	50	300	30	1.722
10	2:1	15	40	200	20	1.188
11	3:1	15	40	300	20	1.768
12	2:1	10	40	200	30	1.298
13	3:1	20	40	300	30	1.687
14	1:1	15	40	200	30	1.362
15	2:1	10	30	300	30	1.381
16	1:1	15	40	400	30	1.452
17	2:1	15	40	300	30	2.145
18	2:1	15	30	400	30	1.630
19	2:1	20	40	200	30	1.660
20	2:1	15	30	200	30	1.359
21	3:1	15	50	300	30	1.995
22	2:1	10	50	300	30	1.548
23	3:1	15	40	200	30	1.591
24	1:1	15	50	300	30	1.998
25	2:1	10	40	300	20	1.781
26	2:1	20	40	300	40	2.091
27	2:1	10	40	300	40	1.900
28	2:1	15	40	300	30	2.193
29	1:1	15	40	300	40	1.896
30	1:1	15	40	300	20	1.575
31	2:1	15	30	300	40	1.730
32	2:1	15	40	400	40	1.812
33	2:1	20	30	300	30	1.424
34	2:1	20	40	300	20	1.700
35	3:1	15	30	300	30	1.481
36	2:1	15	40	300	30	2.039
37	2:1	15	40	300	30	2.340
38	1:1	10	40	300	30	1.211
39	2:1	15	40	300	30	2.183
40	1:1	20	40	300	30	1.870
41	2:1	15	40	200	40	2.251
42	2:1	15	50	200	30	1.990
43	2:1	15	40	400	20	1.931
44	2:1	15	50	400	30	1.519
45	2:1	15	30	300	20	1.610
46	2:1	15	40	300	30	2.259

### Xylitol Production

After hydrolysis under the above optimal conditions, the hydrolysate was filtered and the obtained partial volume was concentrated under vacuum at 70°C to increase the xylose concentration 2–5-fold. The pH of the original and concentrated hydrolysates was adjusted to 5.5 with CaO and then filtered to use as culture media. The fermentation media containing hydrolysates obtained as a function of different treatments, yeast extract (3 g/L), peptone (3 g/L), (NH_4_)_2_SO_4_ (10 g/L), KH_2_PO_4_ (2.4 g/L), MgSO_4_ (0.2 g/L), and CaCl_2_ (0.3 g/L) were autoclaved at 115°C for 15 min. Then, pre-cultured *Candida tropicalis* was inoculated in a sterile flask containing 50 mL of fermentation medium under agitation with 200 rpm at 30°C until the end of fermentation. After incubation, the fermentation media was centrifuged and the supernatant was carefully collected. The xylitol content in the supernatant solutions was measured using the colorimetric assay method with a slight modification (21). The xylitol yield was calculated by the following Equation (1):


$$\begin{array}{l} \text{Xylitol yield }=\;\left(\text{Xylitol content}/\text{Initial xylose content}\right)\times 100 \end{array}$$

## Results and Discussion

### Effects of Ultrasonic-Assisted Pretreatment of NaOH and Its Mixed Alkaline Solution on Enzymatic Hydrolysis of Sugarcane Bagasse

**Figure 1:**
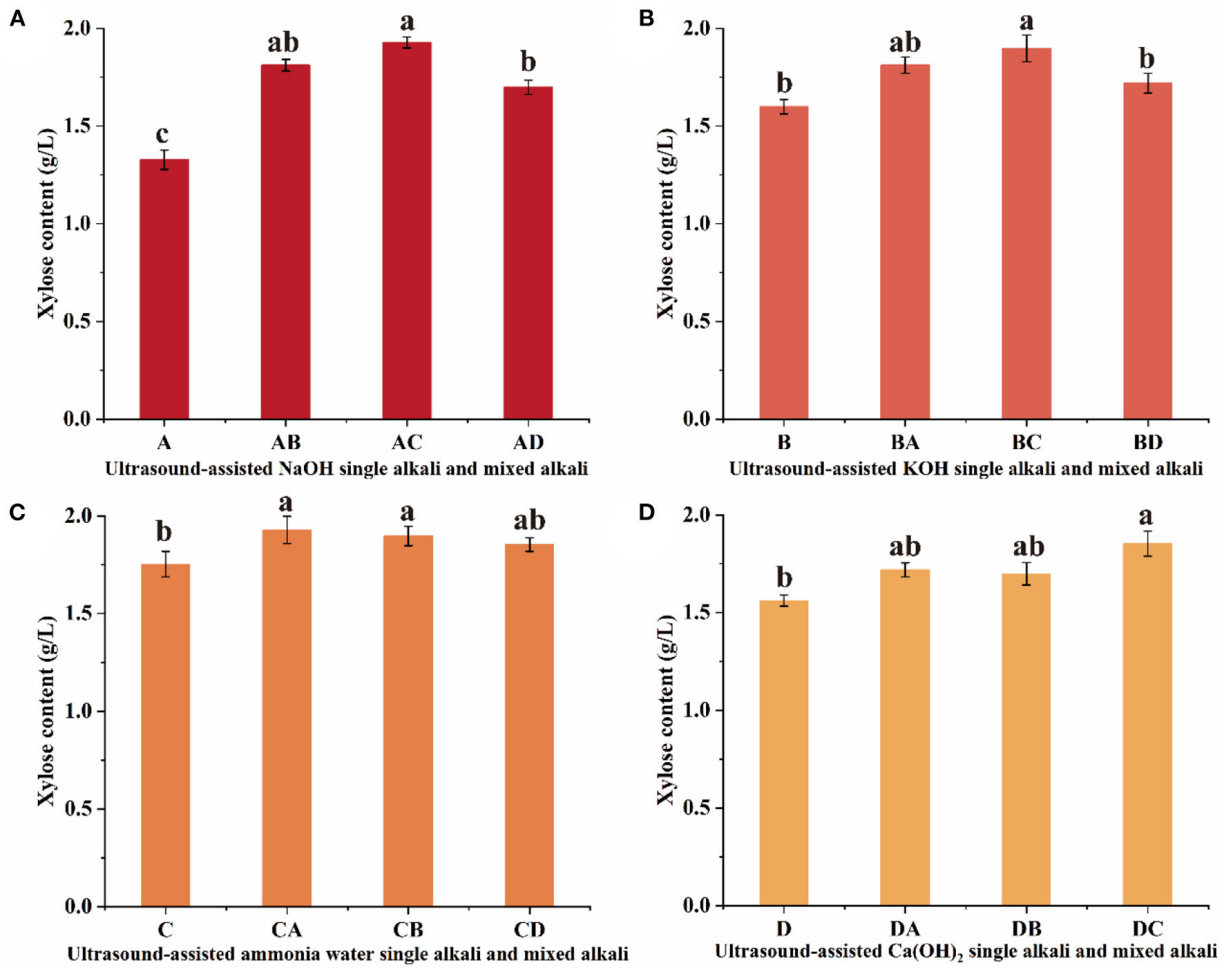
Effects of ultrasonic-assisted **(A)** NaOH, **(B)** KOH, **(C)** ammonia water, and **(D)** Ca(OH)_2_ and its mixed alkaline solution on xylose content from sugarcane bagasse enzymatic hydrolysate. Error bars are the standard deviation of three replications and different lowercase letters (a–c) on top of the bars indicate a significant difference (*P* < 0.05). Different letters are significantly different (*P* < 0.05).

The xylose content produced by ultrasound-assisted mixed alkali pretreatment was higher than which obtained in NaOH single alkali pretreatment, and the xylose content was AC >AB > AD (Figure 1A). The xylose content was 1.928 g/L for mixed alkaline solution treatment at AC, which was significantly (*P* < 0.05) increased by 45% compared to the NaOH single alkali pretreatment. Several factors have been identified to affect the enzymatic hydrolysis of sugarcane bagasse, which include accessible surface area, crystallinity, and polymerization degree of lignocellulose (22). Pretreatment is the process to alter indirect factors and improve direct factors, thus enhancing the accessibility of cellulose. The results on the NaOH and its mixed alkaline treatment of sugarcane bagasse proved that the treatment modifies the composition, structure, and properties of the fibers. Hemicellulose and lignin content decrease, while cellulose crystals disintegrate and dissolve. The sodium ions treatment can loosen up the structure of lignocellulose (23). Lignocellulose structural degradation induced by ultrasonic treatment is attributed to cavitation effects, and reduces its surface area, thus enhancing the subsequent enzymatic hydrolysis rate.

In addition, the ammonia water pretreatment can remove lignin and inhibit acetic acid produced by fermentation, providing favorable conditions for microbial fermentation of xylitol (24). An example of this is the study carried out by Tizazu et al. (25) in which a xylitol yield of 0.66 g/g was obtained in ultrasound-assisted from sugarcane bagasse using immobilized *Candida tropicalis*. Another example by Chen et al. (26) was the xylose recovery of 55.54% and ethanol fermentation efficiency of 93.37% from sugarcane bagasse by two-stage ultrasonic-assisted dilute acid.

### Effects of Ultrasonic-Assisted Pretreatment of KOH and Its Mixed Alkaline Solution on Enzymatic Hydrolysis of Sugarcane Bagasse

The xylose content produced by ultrasound-assisted mixed alkali pretreatment was higher than that of KOH single alkali pretreatment, and the xylose content was BC > BA > BD (Figure 1B). It is worth stressing that the xylose content of BC was significantly increased by 19% compared to B (*P* < 0.05), but the xylose content was not significantly different between the KOH mixed alkali treatments (*P* > 0.05). Alkaline is an effective reagent for delignification from plants, and alkali pretreatments can increase cellulose digestibility, and they are impressive for lignin solubilization, exhibiting minor cellulose and hemicellulose solubilization. KOH is a strong alkali solution, and hydroxyl ion can destroy the tight structure formed between hemicellulose, lignin, and cellulose, increasing the accessibility of xylanase to hemicellulose (27). Compared to NaOH mixed alkali pretreatment, the growth rate of xylose content with KOH pretreatment did not increase significantly, and the market price of KOH was more expensive, which was not favorable for further fermentation to produce xylitol. In a similar case, Safirzadeh (28) compared the pretreatment of sugarcane bagasse with different concentrations of NaOH and KOH. They found that NaOH was more effective than KOH in reducing the lignin and hemicellulose contents.

### Effects of Ultrasonic-Assisted Pretreatment of Ammonia Water and Its Mixed Alkaline Solution on Enzymatic Hydrolysis of Sugarcane Bagasse

The xylose content produced by ultrasound-assisted mixed alkali pretreatment was higher than that of ammonia water single alkali pretreatment, but there was no significant difference in xylose content between the mixed ammonia water pretreatments (*P* > 0.05) (Figure 1C). Ammonia water pretreatment is an alkali pretreatment method and has been widely used in hemicellulase research (29). The pretreatment of ammonia water fiber expansion resulted in cellulose decrystallization, partial hemicellulose depolymerization, lignin C–O–C bond cleavage, and increased accessible surface area due to structural disruption (30). In addition, ammonia water is cheap, volatile, and can be recycled and reused, so it is an ideal method for pretreatment of sugarcane bagasse enzymatic. It can be seen that ultrasonic-assisted NaOH and ammonia water mixed alkali pretreatment resulted in higher xylose content. Wang et al. (31) obtained a total reducing sugar concentration of 16.23 g/L through NaOH and ammonia water pretreatment of enzymatic hydrolysis from sugarcane bagasse.

### Effects of Ultrasonic-Assisted Pretreatment of Ca(OH)_2_ and Its Mixed Alkaline Solution on Enzymatic Hydrolysis of Sugarcane Bagasse

The xylose content produced by ultrasound-assisted mixed alkali pretreatment was higher than that of Ca(OH)_2_ single alkali pretreatment, and the xylose content was DC > DB > DA (Figure 1D). Notably, the xylose content was significantly increased by 18% in the Ca(OH)_2_ mixed alkaline solution treatment compared with the Ca(OH)_2_ single alkaline solution treatment (*P* < 0.05). NaOH pretreatment particularly cleaves the ester bonds in lignin-carbohydrate complexes, and the carbon-to-carbon bonds in lignin molecules, while Ca(OH)_2_ pretreatment mainly removes acetyl groups (32). Additionally, Ca(OH)_2_ belongs to a binary strong base and the hydroxyl ion can destroy the tight structure formed between hemicellulose. Ca(OH)_2_ was slightly soluble in water, which made the solid-liquid separation of enzymatic hydrolysate difficult and unfavorable for further microbial fermentation of xylitol. NaOH has a moderate cost in comparison with KOH and Ca(OH)_2_. Similarly, Chang et al. (33) reported that NaOH pretreatment was more suitable for treating sugarcane bagasse than Ca(OH)_2_ pretreatment due to its advantage of more significant enzymatic hydrolysis efficiency and easier lignin recovery.

### Structural Characterization of Sugarcane Bagasse

**Figure 2:**
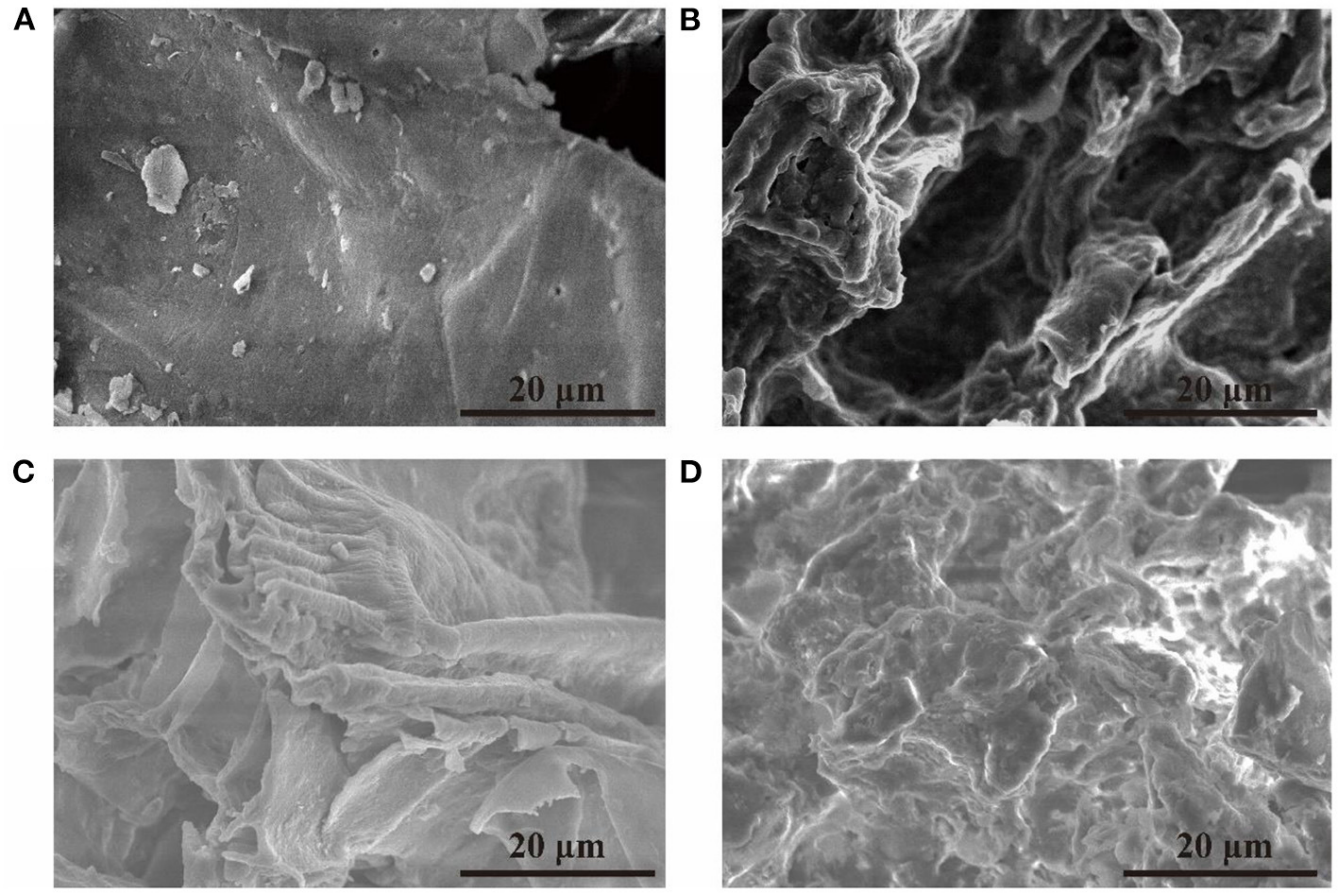
The SEM images of sugarcane bagasse with different pretreatments. **(A)** Without pretreatment, **(B)** with ultrasonic-assisted single alkali pretreatment, **(C)** with ultrasonic-assisted mixed alkali pretreatment, **(D)** with ultrasonic–assisted mixed alkali pretreatment and enzymatic hydrolysis.

The surface morphology of the sugarcane bagasse was demonstrated by the SEM micrographs (Figure 2). The untreated sugarcane bagasse had a smooth and continuous surface, which was not conducive to the enzymatic hydrolysis process (Figure 2A). Rough surface (Figure 2B) and even larger holes (Figure 2C) could be observed after the treatment with solutions of single and mixed alkali, and the fibers lost their integrity after the treatment with enzymatic hydrolysis (Figure 2D). These observations demonstrated that ultrasonic-assisted mixed alkali treatment had a significant effect on the structure of sugarcane bagasse, and cellulose became more accessible to enzymes. Similar structural changes were reported earlier for sugarcane bagasse pretreated with short duration microwave (34) and rice straw pretreated with ammonia water soaking pretreatment (35).

### Optimization of Xylose Content by Box–Behnken Design

From the experimental conditions and corresponding response values shown in Table 1, the experimental data were analyzed by multiple regression. The xylose content was predicted by the second-order polynomial Equation (2), as follows:


$$\begin{array}{l} y=2.19\;+\;0.034A\;+\;0.09B\;+\;0.18C\;-0.025D\;+\;0.13E\\ \;\begin{array}{l} -\;0.14AB\;+\;0.0005AC\;-\;0.078AD\;-\;0.041AE\;+\;0.033BC\\ -\;0.092BD\;+\;0.068BE\;-\;0.19CD\;-\;0.013CE\;-\;0.30DE\\ -0.30A^{2}-0.36B^{2}-0.23C^{2}-0.40D^{2}-0.036E^{2} \end{array} \end{array}$$

where Y is the xylose content; A, B, C, D, and E represent the ratio of NaOH and ammonia water, mixed alkali concentration (%), ultrasonic temperature (°C), ultrasonic power (W), and ultrasonic time (min), respectively.

The analysis of variance (ANOVA) for the response surface quadratic model was summarized in Table 2. The ideal regression equation and the high model significance were confirmed by its high *F* value (11.0712) and low *P*-value (*P* < 0.0001). The goodness-of-fit and adequacy were testified by the determination coefficient (R^2^ = 0.8985) and adjusted determination coefficient (${\text{R}}_{\text{adj}}^{2}$ = 0.8174), respectively. There was no significance in the lack of fit (*P* > 0.05) in the model (*P*-value 0.2351), which indicates the accurate prediction of the model response. As summarized in Table 2, the reaction factors of B (*P* = 0.0136 < 0.05), C (*P* < 0.0001), E (*P* = 0.0006 < 0.01), AB (*P* = 0.0493 < 0.05), CD (*P* = 0.0114 < 0.05), DE (*P* = 0.0002 < 0.01), A^2^, B^2^, C^2^, and D^2^ (*P* < 0.0001) were significant in this model.

**Table 2:** Regression coefficients estimate and significance test for the quadratic polynomial model.

**Source**	**Sum of squares**	**df**	**Mean squares**	***F*** **-value**	***P*** **-value**	
Model	4.0678	20	0.2034	11.0712	<0.0001	Significant
A	0.0189	1	0.0189	1.0314	0.3196	
B	0.1294	1	0.1294	7.0447	0.0136	Significant
C	0.5196	1	0.5196	28.2847	<0.0001	Significant
D	0.0103	1	0.0103	0.5602	0.4612	
E	0.2807	1	0.2807	15.2816	0.0006	Significant
AB	0.0784	1	0.0784	4.2691	0.0493	Significant
AC	0.0000	1	0.0000	0.0001	0.9924	
AD	0.0246	1	0.0246	1.3392	0.2581	
AE	0.0068	1	0.0068	0.3696	0.5487	
BC	0.0043	1	0.0043	0.2357	0.6316	
BD	0.0341	1	0.0341	1.8549	0.1854	
BE	0.0185	1	0.0185	1.0046	0.3258	
CD	0.1372	1	0.1372	7.4680	0.0114	Significant
CE	0.0007	1	0.0007	0.0354	0.8523	
DE	0.3496	1	0.3496	19.0285	0.0002	Significant
A2	0.7637	1	0.7637	41.5705	<0.0001	Significant
B2	1.1509	1	1.1509	62.6487	<0.0001	Significant
C2	0.4468	1	0.4468	24.3210	<0.0001	Significant
D2	1.4025	1	1.4025	76.3442	<0.0001	Significant
E2	0.0114	1	0.0114	0.6191	0.4388	
Residual	0.4593	25	0.0184			
Lack of Fit	0.4073	20	0.0204	1.9579	0.2351	Not significant
Pure Error	0.0520	5	0.0104			
Cor Total	4.5271	45				
Adeq Precision	10.9710					
R^2^	0.8985					
${\text{R}}_{\text{Pred}}^{2}$	0.6236					
${\text{R}}_{\text{ADj}}^{2}$	0.8174					

*The df represents degree of freedom*.

*Significant coefficient (P < 0.05)*.

**Figure 3:**
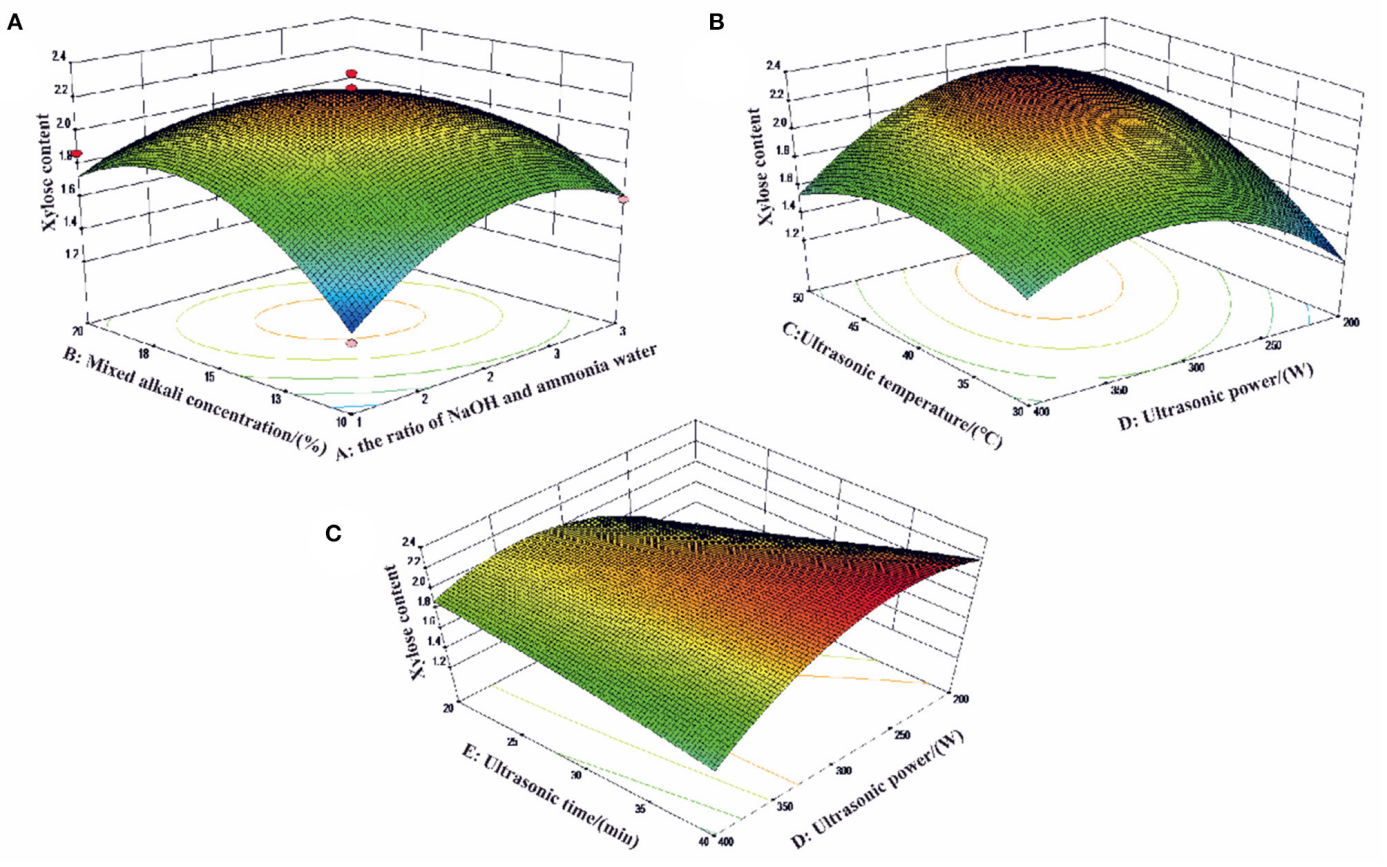
The effects of **(A)** the ratio of NaOH and ammonia water; **(B)** mixed alkali concentration (%); **(C)** ultrasonic temperature (°C); (D) ultrasonic power (W) and (E) ultrasonic time (min) on xylose content of three-dimensional (3D) response surface curves.

The 3D response plot shown in Figure 3 were graphical representations of the regression equation. The comprehensive effects of the ratio of NaOH and ammonia water (A), mixed alkali concentration (B), ultrasonic temperature (C) ultrasonic power (D), and ultrasonic time (E) on xylose content were discussed and the optimal condition for xylose content was proposed as well. Figure 3A shows the combined effect of mixed alkali concentration (B) and the ratio of NaOH and ammonia water (A) on xylose content. It indicated that the xylose content decreased at first and then increased with the increasing mixed alkali concentration and the ratio of NaOH and ammonia water. Quadratic effects of ultrasonic temperature (C) and ultrasonic power (D) were shown in Figure 3B. The xylose content increased when ultrasonic power increased from 200 to 300 W. Even though higher ultrasonic power yielded higher xylose content, more thermal energy was generated resulting in the increasing reaction temperature from 30 to 40°C. Therefore, the ultrasonic power in this study was controlled at a moderate degree of 250–350 W to avoid cellulose structure regrouping and improve the xylose content as well. Figure 3C showed that the ultrasonic power (D) and ultrasonic time (E) had a quadratic effect on the xylose content. The xylose content decreased with increasing reaction time.

In summary, when the ratio of NaOH and ammonia water was 2:1, the mixed alkali concentration (v/v) was 17%, the ultrasonic temperature was 45°C, the ultrasonic power was 300 W, and the ultrasonic time was 40 min, the maximum content of xylose in the actual experiment (2.431 g/L) was obtained, and this value was consistent with the theoretically predicted value (2.462 g/L).

### Xylitol Production From Sugarcane Bagasse Hydrolysate by *Candida tropicalis*

**Figure 4:**
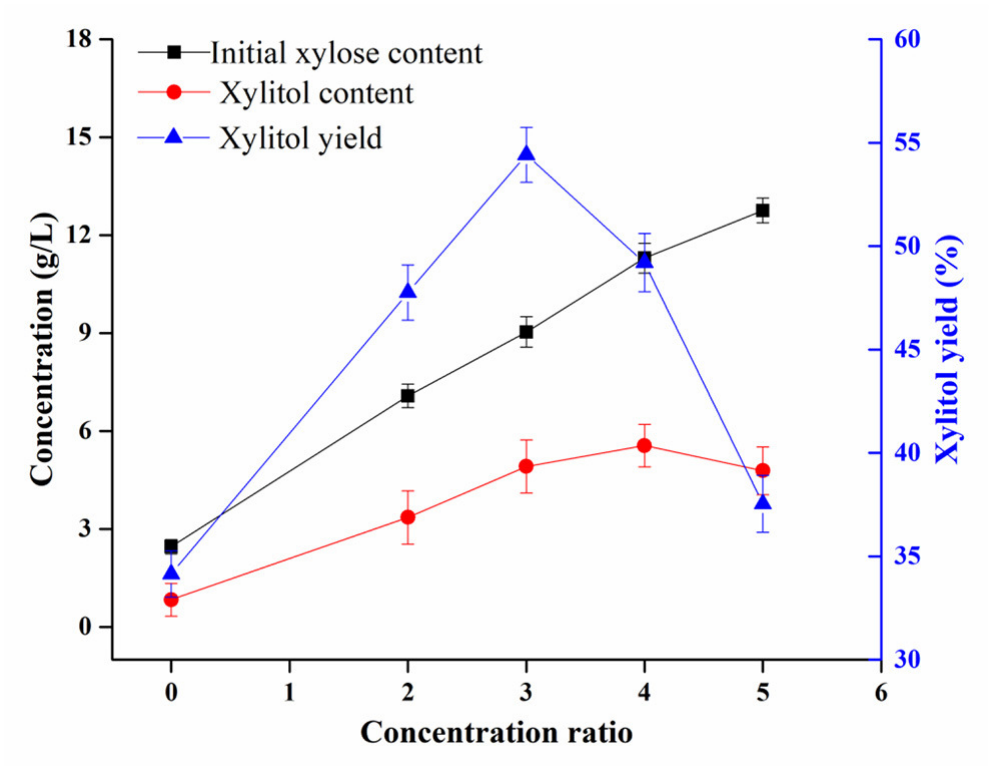
Influence of the concentration ratio of the enzymatic hydrolysate of sugarcane bagasse on xylitol obtained by fermentation with *Candida tropicalis*.

The production of xylitol by fermentation of *Candida tropicalis* based on the sugarcane bagasse hydrolysate obtained by the above method was investigated. As shown in Figure 4, the content of xylitol obtained in the original hydrolysate was 0.84 g/L. With the increase in the concentration ratio of the hydrolysate, the obtained xylitol content gradually increased and the maximum value reached 5.56 g/L. However, this value decreased when the concentration ratio exceeded 4 times. By contrast, a similar tendency was obtained for xylitol yields. The yield of xylitol obtained in the original hydrolysate was 34.15%. As the concentration ratio of the hydrolysate increased, the obtained xylitol yield gradually increased and the maximum value reached 54.42%. However, this value decreased when the concentration ratio exceeded 3 times. These results are in agreement with the previous study that the concentration ratio of the sugarcane bagasse hydrolysate significantly affected the xylose reductase and xylitol dehydrogenase activity of yeast which may lead to differences in xylitol yields (36). Moreover, previous studies reported that fermentation inhibitors such as acetic acid and furfural were present in the bagasse hydrolysate (36, 37). In this case, the xylitol fermentation was severely inhibited, resulting in a low yield of xylitol. With the increase of the concentration ratio, the yield of xylitol increased gradually due to the reduction of volatile harmful substances in the hydrolysate. In addition, when the xylose concentration was low, *Candida tropicalis* mainly use xylose for its growth, which may also lead to low xylitol yield. When the hydrolysate was concentrated to a certain multiple, the xylose concentration gradually approached the most favorable value for fermentation, which increased the yield of xylitol. However, when the concentration ratio exceeded 3 times, the yield of xylitol decreased due to the loss of xylose, the formation of fermentation inhibitors and the accumulation of non-volatile fermentation inhibitors in the hydrolysate. Therefore, the sugarcane bagasse hydrolysate used for xylitol fermentation should be reasonably concentrated and further detoxified.

## Conclusions

In this study, xylitol production from sugarcane bagasse through ultrasonic-assisted dual-alkali pretreatment and enzymatic hydrolysis followed by *Candida tropicalis* fermentation were investigated. First, the effects of ultrasonic-assisted NaOH, KOH, ammonia water, Ca(OH)_2_ and their mixed double-alkali solution pretreatment on the enzymatic hydrolysis of sugarcane bagasse were compared. The results showed that the highest xylose content (1.928 g/L) was obtained under the condition of ultrasonic-assisted NaOH and ammonia water mixed alkali pretreatment. Then, the processing parameters were optimized by RSM coupled with BBD. When the ratio of NaOH and ammonia water was 2:1, the mixed alkali concentration (v/v) was 17%, the ultrasonic temperature was 45°C, the ultrasonic power was 300 W, and the ultrasonic time was 40 min, the content of xylose reached a maximum of 2.431 g/L. The sugarcane bagasse pretreated with ultrasonic-assisted alkaline pretreatment had more folds and furrows in external appearance. Moreover, the results of *Candida tropicalis* fermentation showed that the yield of xylitol increased (maximum 54.42%) with the increase of the concentration ratio of the enzymatic hydrolysate, but this value decreased when the concentration ratio exceeded 3 times. Overall, this study provides a high-value utilization of sugarcane bagasse for xylitol production.

## Data Availability Statement

The raw data supporting the conclusions of this article will be made available by the authors, without undue reservation.

## Author Contributions

LG: writing—original draft, software, formal analysis, and visualization. E-FR: conceptualization, data curation, and writing—review and editing. WT: investigation, resources, and writing—original draft. DN: funding acquisition, supervision, writing—review and editing, resources, and validation. WS: investigation, supervision, writing—review and editing, and project administration. FH and KL: writing—review and editing. All authors contributed to the article and approved the submitted version.

## Funding

This work was supported by the National Natural Science Foundation of China (32102133) and the Natural Science Foundation of Guangxi Province (2021JJA130374).

## Conflict of Interest

The authors declare that the research was conducted in the absence of any commercial or financial relationships that could be construed as a potential conflict of interest.

## Publisher's Note

All claims expressed in this article are solely those of the authors and do not necessarily represent those of their affiliated organizations, or those of the publisher, the editors and the reviewers. Any product that may be evaluated in this article, or claim that may be made by its manufacturer, is not guaranteed or endorsed by the publisher.
